# Clinical-Pathological Conference Series from the Medical University of Graz: Case No 166: An 82-year-old woman with voluminous diarrhea and weight loss

**DOI:** 10.1007/s00508-022-02112-0

**Published:** 2022-12-19

**Authors:** Elisabeth Fabian, Thomas Roskaric, Johann Pfeifer, Heimo Wenzl, Heinz F. Hammer, Carolin Lackner, Georg Rosanelli, Guenter J. Krejs

**Affiliations:** 1 Department of Internal Medicine II , University Hospital Krems, Karl Landsteiner University of Health Sciences, Krems on the Danube, Austria; 2 Department of Surgery, State Hospital Wolfsberg, Wolfsberg, Austria; 3 Department of Surgery, Medical University of Graz, Graz, Austria; 4 Division of Gastroenterology and Hepatology, Department of Internal Medicine, Medical University of Graz, Auenbruggerplatz 15, 8036 Graz, Austria; 5 Department of Pathology, Medical University of Graz, Graz, Austria; 6 Department of Surgery, Elisabethinen Hospital, Graz, Austria

**Keywords:** Enterocolonic fistula, Colon diverticulitis, Fistulectomy, Anastomotic leak, Breath tests

## Presentation of case

*Dr. T. Roskaric*: Six years prior to admission, the patient underwent surgery for an inflammatory conglomerate tumor due to complicated sigmoid diverticulitis; 28 cm of the colon and 18 cm of the small bowel were resected. After surgery, the patient was free of symptoms and passed 2–3 stools per day. Six months before admission, she developed watery to mushy diarrhea with 15–20 bowel movements per day but without abdominal pain. The symptoms became worse after eating and the patient reported food residues in her stool. Since the onset of these complaints, the patient has lost about 20 kg of body weight (current body weight: 62 kg, body height: 164 cm, body mass index: 23 kg/m^2^).

A comprehensive assessment of her condition in another hospital revealed abnormal breath tests suggesting lactose intolerance, fructose malabsorption and small intestinal bacterial overgrowth (SIBO). Esophagogastroduodenoscopy was unremarkable as was the histologic examination of the duodenal biopsies. Colonoscopy showed considerable inflammation of the colorectal anastomosis and the terminal ileum. Histology of the anastomosis found inflammation, extensive ulcers and granulation tissue as well as small bowel mucosa.

Laboratory: Hemoglobin 10.0 g/dL (normal: 14.0–17.5 g/dL), mean corpuscular volume (MCV) 91 fL (normal: 80–98 fL), serum iron 42 μg/dL (normal: 37–145 µg/dL), ferritin 240 ng/mL (normal: 13–150 ng/mL), serum levels of vitamin B_12_ and folate were normal. For her normocytic anemia, which was due to chronic kidney disease (CKD) of cardiovascular origin, she received erythropoietin.

During the last 6 months, the patient’s diarrhea was unsuccessfully treated with different antibiotics including rifaximin. Repeated stool cultures were without pathologic findings. Administration of 5‑aminosalicylic acid had no beneficial effect. Treatment with loperamide and budesonide resulted in a slight transient improvement, but a permanent relief of symptoms has not been achieved. A therapeutic trial with cholestyramine markedly reduced her diarrhea but had to be terminated because of nausea.

The patient suffers from multiple comorbidities such as coronary heart disease, arterial hypertension, type 2 diabetes mellitus, chronic kidney disease (CKD) and osteoporosis, and has undergone multiple surgeries (thyroidectomy, hip and knee replacement, and osteosynthetic stabilization of the lumbar spine). Her medication includes acetylsalicylic acide, nicorandil, losartan, a calcium channel blocker and a beta-blocker, a proton pump inhibitor, levothyroxine and mirtazapine.

On admission, the patient reported between 14 and 22 bowel movements (about 1.5 L mushy stool) within 24 h during the last 4 days. Stool cultures were negative for enteric pathogens, calprotectin in stool was 240 mg/kg (normal: < 50 mg/kg). Laboratory data showed a creatinine level of 2.8 mg/dL (normal: < 1 mg/dL) and a glomerular filtration rate of 21 mL/min/1.73 m^2^ (normal: 90–120 mL/min/1.73 m^2^); serum electrolytes were within normal limits, albumin 4.0 g/dL (normal: 3.5–5.2 g/dL), C‑reactive protein (CRP) 25.9 mg/dL (normal: < 5 mg/dL).

Colonoscopy showed similar findings as reported earlier and was additionally suggestive of antibiotic-associated colitis in the distal colon. Histology of several biopsies described reactive inflammation of colonic mucosa and normal small bowel mucosa.

Abdominopelvic computed tomography (CT) with rectal administration of 150 mL of a contrast medium could not be assessed adequately due to artifacts caused by metal implanted during the past surgeries. Contrast medium reached up to the level of the anastomosis in front of the sacrum; differentiation between small bowel and colon was not possible because of the mentioned artifacts.

A diagnostic test was performed.

## Differential diagnosis

*Dr. J. Pfeifer:* This is an interesting case of an elderly woman who presented with a 6-month history of diarrhea and weight loss. She reported an increase in bowel movements after eating and food residues in stool, but she denied abdominal pain. Her medical history was positive for abdominal surgery of an inflammatory conglomerate tumor due to complicated diverticulitis with resection of parts of the colon and the small bowel 6 years earlier.

Diverticular disease is a common gastrointestinal problem in western countries, especially in patients of advanced age [1]. In the USA (population of 340 million), it accounts for nearly 2.7 million outpatient visits and about 370,000 presentations in emergency departments per year [2]. The US data further show about 208,000 hospital admissions per year due to diverticular disease and about 150,000 emergency admissions because of acute diverticulitis, the inflammatory complication of this disease [3]. About 20–30% of admitted patients with diverticulitis present with complications such as abscess, perforation, peritonitis, obstruction or fistula; two thirds show uncomplicated disease, which is frequently treated with antibiotics [4–8]. The prevalence of diverticular disease is age-dependent and increases from less than 20% at age 40 years to 60% by age 60 years[9]. The majority of patients (95%) with diverticula have sigmoid diverticula [10] and in about 65% of patients they are limited only to the sigmoid colon [11]. As the prevalence of diverticulosis increases with age, the incidence of diverticulitis increases as well. The mean age of patients with acute diverticulitis on admission is 63 years [12]. In contrast to Asia, in western countries diverticular disease is predominantly left-sided and only 1.5% of cases present with right-sided diverticulitis [13]. In patients under 50 years of age, diverticulitis is more common in men, but with increasing age this changes to a marked preponderance of women [9]. About 20% of patients with acute diverticulitis require emergency surgery [14]. Sigmoid colectomy with primary colorectal anastomosis with or without proximal diversion is reserved for hemodynamically stable patients, whereas an end-colostomy (Hartmann procedure) is the preferred surgical intervention for those who are unstable or in septic shock [15]. In summary, surgery for acute diverticulitis typically results in colorectal anastomosis; resection of the small bowel as documented in the discussed patient is not common, but may have been necessary due to intraoperative complications. Thus, the histologic findings of the presence of small bowel mucosa at the anastomosis is atypical and cannot be explained by the patient’s prior surgery; however, the histologic finding of inflammation, extensive ulcers and granulation tissue in this patient suggests a chronic inflammatory process at the anastomosis.

Abdominal pain is the hallmark of acute diverticulitis and even after surgery about 25% of patients have ongoing symptoms [16]. In some patients there is disease progression or recurrence even after surgery [17]. Indeed, initial presentation of complicated diverticulitis is a well-established risk factor for recurrent episodes of diverticulitis [15]. Several retrospective studies reported a recurrence rate between 24% and 60% in these patients [18–20]. During a median follow-up of 3.9 years, patients with a history of complicated diverticulitis were more frequently readmitted (12%) than patients who had initially presented with uncomplicated disease (8%) [21]. Moreover, data show that patients with complicated diverticulitis were more likely to experience a recurrent episode at any time up to 14 years after the initial presentation [22]. Thus, affected patients should receive counseling regarding the increased risk of recurrence and possible complications [15]. Another potential risk factor associated with recurrence of diverticulitis is an involved colon length greater than 5 cm and the presence of a retroperitoneal abscess [23] (in our patient, the initially affected colon segment was 28 cm in length). In addition, the maximum wall thickness in the inflamed segment and the severity of inflammation on abdominopelvic CT scan are possible predictors for recurrent diverticulitis [24]. In the discussed patient, the complicated disease on initial presentation and an affected length of the colon greater than 5 cm, are regarded as factors that could have predisposed to future complications; however, in this case, no abdominal complaints except for excessive diarrhea were reported. The absence of abdominal pain makes the diagnosis of progressive or recurrent diverticulitis unlikely.

Given the patient’s advanced age and history of coronary heart disease and anemia, one may think of ischemic colitis in this patient; however, this diagnosis can also be ruled out because the patient denied abdominal pain. Furthermore, malabsorptive diseases such as celiac disease can be excluded as well on the basis of normal histology of duodenal biopsies. Although positive breath tests suggested lactose intolerance, fructose malabsorption and SIBO, these diagnoses are questionable as positive test results may also be obtained due to accelerated gastrointestinal transit. In addition, the absence of symptoms such as abdominal pain and bloating, which are typically found in patients with lactose intolerance, fructose malabsorption or SIBO, make these diagnoses unlikely in this case.

Since the patient’s history was negative for travel abroad, and repeated stool cultures were negative for pathogens, an infectious disease also seems unlikely in this case. Excessive high-volume diarrhea is a hallmark of the Verner-Morrison syndrome, also known as watery diarrhea, hypokalemia, hypochlorhydria (WDHH) syndrome [25]; however, laboratory data showed normal serum electrolytes, which excludes this diagnosis in the reported case.

As a therapeutic trial with cholestyramine was positive, causes of cholorrheic diarrhea such as (1) escape of dihydroxy bile acids from absorption and subsequent stimulation of colonic secretion, (2) extensive resection of the ileum and (3) the blind loop syndrome should be considered; however, resection of only 18 cm of the ileum usually does not result in bile acid-induced diarrhea and the patient history is negative for surgery resulting in a blind loop or ileocolonic anastomosis. Thus, there must have been an alternative mechanism which led to a connection between the colon and the small bowel. Given this anatomic constellation and the histologic evidence of reactive inflammation of the anastomotic site as well as normal small bowel mucosa which the gastroenterologist did not know how to interpret, strongly suggests the formation of a fistula in this patient. Thus, in view of the entire constellation of findings in this case, diarrhea caused by a shortened gastrointestinal transit due to an enterocolonic fistula seems to be the most likely diagnosis. This should be confirmed radiographically with contrast medium introduced either from above (small bowel follow through) or from below (barium enema).

## Dr. J. Pfeifer’s diagnosis

Enterocolonic fistula

## Discussion of case

*Dr. C. Lackner: *Histology of the biopsies taken during colonoscopy did not correlate with the clinical data which reported incomplete colonoscopy, stating “terminal ileum had not been reached”. Out of seven samples six revealed normal small bowel mucosa without any pathologic findings. Only one biopsy showed mucosa of the colon with signs of reactive inflammation but was otherwise unremarkable.

**Fig. 1:**
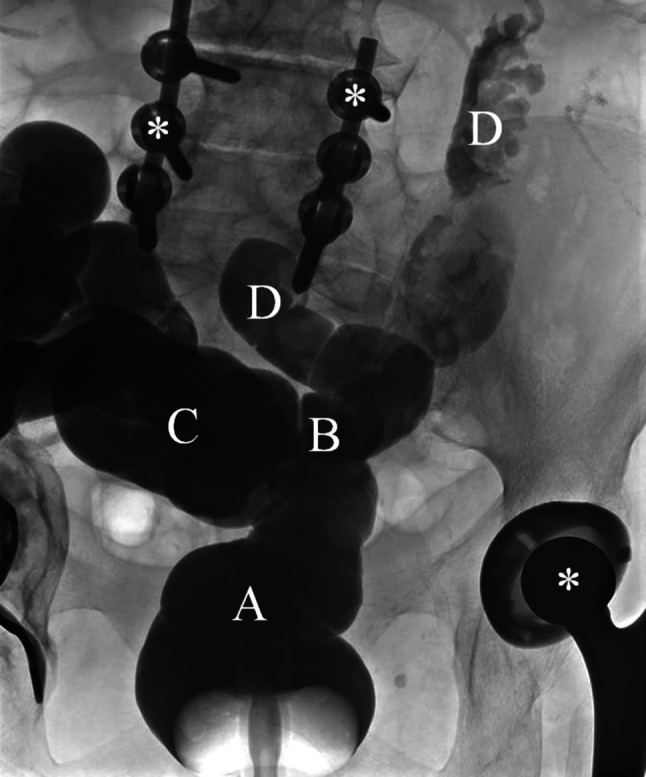
Barium enema performed with special attention in search of an abnormal connection between the colon and small intestine: *A* rectum, *B* fistula connecting the proximal rectum and small bowel, *C* descending colon, *D* small bowel, *asterisks*: metal from hip replacement and lumbosacral stabilization which caused artifacts on CT scan

*Dr. H. Wenzl:* The remarkable discrepancy between the endoscopy report and the histologic findings prompted us to perform a barium enema in order to clarify the postoperative anatomy in our patient. The investigation revealed an enterocolonic fistula adjacent to the colorectal anastomosis (Fig. 1), which had apparently developed during the last 6 months due to ongoing inflammation after surgery for complicated diverticulitis 6 years earlier.

**Fig. 2:**
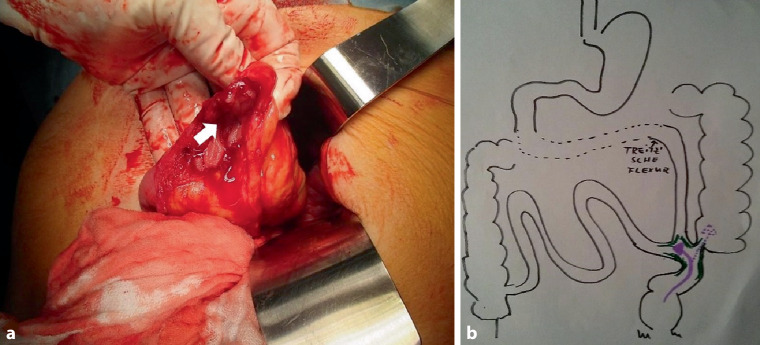
Operative findings: The surgeon (G.R.) is holding the small bowel segment that was separated from the colon after resection of the fistula. One can see the small bowel open where the fistula entered (*arrow*) (**a**). Immediate postoperative illustration by the surgeon of the location of the fistula (*arrow*) between jejunum and the distal colon (**b**)

*Dr. T. Roskaric: *In the further course, the patient underwent surgery with resection of the fistula without complications. Intraoperatively, the fistula appeared to be located between the lower jejunum and the colon adjacent to the colorectal anastomosis (Fig. 2). After surgery, the patient was free of complaints, did not experience diarrhea anymore and started to gain weight.

**Fig. 3:**
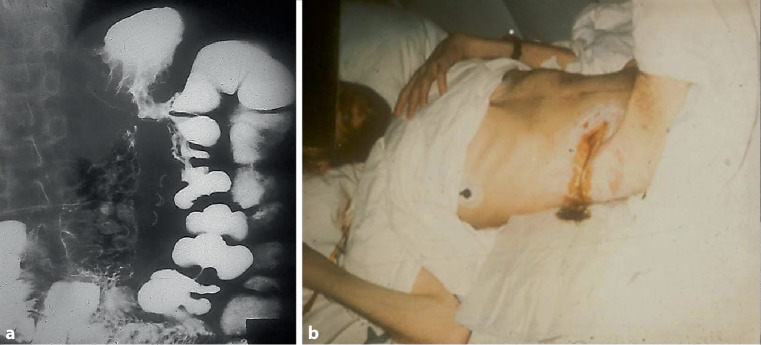
Barium swallow showing a gastrocolonic fistula (**a**). Patient with Crohn’s disease and an enterocutaneous fistula with stool leakage (**b**)

*Dr. G. J. Krejs: *Symptoms of gastrointestinal fistulas are variable and depend on their location. Fistulas that connect the colon to another part of the intestine may result in osmotic diarrhea because of insufficient surface for absorption. This condition tends to worsen with eating [26]. Weight loss caused by the ensuing malabsorption is frequently observed in affected patients. In former days, when proton pump inhibitors were not available and *Helicobacter pylori* was still unknown, gastric ulcers were rather common. Secondary to chronic ulcers, some patients developed gastrocolonic fistulas (Fig. 3), which led to a markedly reduced gastrointestinal transit and the finding of undigested food in the stool. Indeed, undigested food in stool is a hallmark of fistulas from the very proximal to the very distal gastrointestinal tract. Evaluation of the patient’s anatomy is a must in such cases.

However, colonic fistulas may also connect the intestine with the skin, the bladder or vagina, and cause abnormal fluid, stool and gas passage. Before the availability of effective treatment, patients with chronic inflammatory bowel disease were prone to such complications (Fig. 3). Rarely, colobronchial fistulas are also observed; Zhao et al. [27] and our team reported such cases [28].

It is noteworthy that in this case two experienced endoscopists had obviously entered the small bowel through the fistula with their endoscope, but failed to recognize that they were in the small intestine and not in the colon. Furthermore, they did not even consider the possibility of a fistula and did not draw any conclusions from the fact that histology revealed small bowel mucosa. Instead, they assumed a mix-up of samples in the pathology department. The presented case should serve as a lesson for endoscopists and gastroenterologists, alerting them to the fact that it is difficult to recognize small bowel mucosa in the setting of a common microbiome of small and large bowel caused by the fistula.

*Dr. H. F. Hammer*: The elevated calprotectin is a nonspecific finding that can be explained by the inflammation at the anastomosis. In general, mild elevations of calprotectin above the normal range have a low specificity for inflammatory bowel disease (IBS), with mean fecal concentrations in irritable bowel syndrome around 85 µg/g and as high as 400 µg/g in diarrhea-predominant IBS [29]. The results of the breath tests (presumably hydrogen breath tests, H_2_BT) were interpreted as indicating lactose intolerance, incomplete fructose malabsorption and SIBO. In the human body, hydrogen (H_2_) is derived exclusively from anaerobic fermentation of carbohydrates by intestinal microbiota [30]. Increased concentrations of this gas in breath after oral ingestion of a fermentable carbohydrate therefore indicate that the substrate has come into contact with saccharolytic bacteria before it was fully absorbed by the small bowel. This is the physiological basis for the detection of malabsorption of carbohydrates like lactose or fructose, SIBO, and for the measurement of orocecal transit time (OCTT) by H_2_ carbohydrate breath tests. In most cases, carbohydrate malabsorption is clinically relevant only if it causes abdominal symptoms (intolerance). A well-performed H_2_BT addresses this limitation by detecting malabsorption (H_2_ increase above a set diagnostic threshold) and confirming the temporal relationship between this objective event and the occurrence of subjective symptoms [31]. The accuracy of H_2_BT is confounded by certain factors. A false positive H_2_BT, often characterized by a rapid increase in the concentration of H_2_ in the breath, can result from poor oral hygiene, SIBO or rapid intestinal transit. Conversely, a false negative H_2_BT result occurs in at least 10% of patients because the colonic microbiome does not produce sufficient H_2_ to be detected by current technology [31, 32]. SIBO is characterized by a wide spectrum of clinical manifestations, ranging from nonspecific abdominal symptoms (e.g. bloating, abdominal discomfort, flatulence) to less frequent severe generalized malabsorption and nutrient deficiency (diarrhea, anemia, deficiency of vitamins and iron, steatorrhea, weight loss). Multiple independent risk factors have been identified for SIBO, including anatomical abnormalities such as small intestinal diverticulosis and postsurgical structural changes. The culture of jejunal aspirate has long been considered the gold standard diagnostic test for SIBO; however, this approach is invasive and difficult to perform. In addition, aspirates from the proximal jejunum lack sensitivity in all but the most severe cases of bacterial overgrowth which extend as far as the upper parts of the small bowel. The H_2_BT has long been used to detect SIBO based on the principle that an increase in H_2_ indicates contact between carbohydrates and bacteria anywhere in the gastrointestinal tract. H_2_BT has become the most widely used test for SIBO in clinical practice [31]. The key limitation of H_2_BT for the diagnosis of SIBO is the high variability in OCTT in health and disease. The simultaneous assessment of OCTT with a scintigraphy technique is useful to distinguish early peaks of H_2_ due to fast OCTT from peaks due to SIBO [31, 33]. In retrospect, however, the fistula explains a positive breath test after ingestion of lactose, fructose and the carbohydrate which was used for detection of SIBO (glucose or lactulose are commonly used). All of these carbohydrates have presumably escaped absorption in the small intestine and come into contact with bacteria, either by entering the remaining colon through the fistula, or by reaching bacteria which had ascended from the colon to the small bowel through the fistula. Regardless of the exact pathophysiological mechanism in this case, it has to be assumed that there was an early rise in breath hydrogen concentration after all of the three ingested carbohydrates which is compatible with an early contact between the carbohydrate and intestinal bacteria.

*Dr. J. Pfeifer:* This case of a patient with an enterocolonic fistula is interesting and instructive for abdominal surgeons and gastroenterologists alike. Since the history was negative for chronic inflammatory bowel disease, I strongly suggest that the fistula adjacent to the site of the anastomosis developed on the basis of a small anastomotic leak, which is one of the most serious postoperative complications in colorectal surgery, leading to a prolonged hospital stay, decreased quality of life, and increased morbidity and mortality [34]. The reported frequency of anastomotic leak in colorectal surgery varies from 2% to 19%, with the highest risk for low rectal anastomosis [35–37]. Intraoperative testing for integrity and vascular perfusion of the anastomosis may reduce the rate of leakage. Today, different techniques are available. For testing anastomotic integrity intraoperative endoscopy with an air leak test, with or without application of blue-colored saline, might be the best method, because it can also reveal other anastomosis-related complications such as bleeding. For testing perfusion, surgeons formerly relied on subjective assessment of the color of the bowel wall, bleeding from the resection margin and on evaluating the palpable pulsations of the mesenteric arteries. Nowadays, objective methods such as indocyanine green fluorescence angiography are available [34]. Furthermore, data suggest that the intestinal microbiome has a significant influence on the development of anastomotic leaks. Perioperative stressors and surgical manipulation lead to an alteration of the gut microbiome with a reduced abundance of commensal bacteria and proliferation of low-abundance γ‑proteobacteria [38]. As healing-promoting species become depleted, pathogens such as *Pseudomonas aeruginosa, Enterococcus faecalis* and *Serratia marcescens* augment and elicit intestinal inflammation by producing collagenases (proteolytic enzymes mediating extracellular matrix degradation and regulating the release of growth factors, chemokines and adhesion proteins [39]). This dysbiosis is suggested to drive the pathogenesis of anastomotic leaks [40, 41].

One can speculate that suboptimal anastomotic perfusion due to (1) a history of cardiovascular disease (which also caused CKD in this patient), (2) ongoing local inflammation (probably a residual disease or recurrence of the former complicated diverticulitis or associated with intestinal dysbiosis) as well as (3) a chronic inflammatory state (reflected by elevated serum CRP levels) may have predisposed this patient to formation of a fistula adjacent to the anastomosis from a small anastomotic leak.

## Final diagnosis

Enterocolonic fistula
